# Voices from the margins: A qualitative study exploring components influencing psychosocial health and wellbeing among gender minority forced migrants

**DOI:** 10.1177/14034948241301874

**Published:** 2024-12-10

**Authors:** Maria Gottvall, Rummage Isaac, Osszián Péter-Szabó, Ronah Ainembabazi, Tommy Carlsson

**Affiliations:** 1 Department of Health Sciences, The Swedish Red Cross University, Sweden; 2 Department of Women’s and Children’s Health, Uppsala University, Sweden

**Keywords:** Forced migration, health services, gender-non-conforming persons, gender minorities, LGBTQ, refugees, transgender persons

## Abstract

**Aims:**

To explore the components that influence health and wellbeing of gender minority forced migrants residing in Sweden.

**Methods:**

Qualitative exploratory study based on semi-structured interviews with gender minority forced migrants recruited through a combination of convenience, purposeful and snowball sampling. Guided by the levels in the social ecological model, transcripts were analysed with systematic text condensation in a collaborative process between experts by lived experience, researchers and clinical psychologist.

**Results:**

Participants expressed resilience and hope about their future. Loneliness was a major issue contributing to health burdens and peer support was highly appreciated. Barriers hindering access to health services and judgemental behaviours among health professionals were described. Affirming support through empathy, trust, safety, confidentiality, continuity and respect was highlighted as essential in health services. While societal openness and safety for gender minority individuals was appreciated, participants faced an uncertain asylum process and unmet basic needs.

**Conclusions:**

Gender minority forced migrants show resilience and appreciate the newfound societal safety. However, they find themselves in the margins of society and encounter various multi-layered challenges. Loneliness is a public health concern that could be addressed through peer support, which is highly desired and valued. Ensuring access to affirming health services should be a prioritized area for researchers, professionals, stakeholders and policy-makers.

## Background

All humans are born equal with the rights to live safely, free from discrimination and torture [1]. However, persons with a gender identity extending beyond societal norms (e.g. transgender and non-binary persons; hereafter referred to as gender minorities) encounter a range of societal disadvantages and marginalization, contributing to health burdens [2]. Repeatedly, research reports that gender minorities experience higher rates of mental health burdens when compared with cisgender counterparts [3,4]. These health disparities may be attributed to minority stress, gender-based victimization, rejection and discrimination [2,5]. In many countries, these persons face oppression and danger, requiring them to migrate to another country [6]. Forced migration is a significant life event impacting the health and wellbeing of millions [7]. The occurrence of psychological distress, including loneliness and social exclusion, is a well-documented public health concern within the wider population of forced migrants [8,9]. Carrying experiences of previous trauma while dealing with new challenges, many forced migrants experience mental health burdens during resettlement [10].

The psychological stressors experienced in the post-migration period among forced migrants may be intensified for those with intersecting minority identities, contributing to further mental health burdens [11]. Intersectionality is a framework that refers to the complex ways that identities intersect to create discrimination, oppression, disadvantages and privilege in society [12]. Based on that framework, gender minority forced migrants may be at risk of experiencing intersecting disadvantages linked to multiple minority statuses of being both a forced migrant and of a gender identity extending beyond societal norms [13].

The Nordic countries, including Sweden, acknowledge fear of persecution based on gender identity and gender expression as a valid reason for asylum. However, there are no reliable national statistics on the number of gender minority asylum seekers. Forced migrants in Sweden, including asylum seekers and undocumented persons, have the right to receive healthcare that cannot be deferred. However, very little is reported about the experiences of support among gender minority forced migrants. While reviews call attention to the need for improved knowledge of mental health burdens among gender minority forced migrants, there is a substantial lack of research based in the Nordic countries [14,15]. The aim of this study was to explore the components that influence health and wellbeing of gender minority forced migrants residing in Sweden. Specifically, we were interested in understanding experiences related to their psychosocial situation, how they perceived support from health services and how they experienced social support.

## Methods

### Study design and setting

This was an exploratory qualitative interview study utilizing public contribution. Study procedures were conducted in collaboration with two forced migrants acting as experts by lived experience involved throughout all parts of the research cycle. The efforts to involve them were made to reach democratized findings grounded in diverse perspectives and expertise. The study was guided by the social ecological model as a theoretical framework [16], seeking to understand components that influence psychosocial health and wellbeing at four levels: (i) intrapersonal, (ii) interpersonal, (iii) organizational (i.e. health services) and (iv) societal. This study was approved by the Swedish Ethics review authority (approval number: 2022-01483-01). Participants provided informed consent and received a gift card of SEK500.

### Recruitment and sample

Participants were recruited through a combination of convenience, purposive and snowball sampling. Advertisements were posted on websites and social media (the website of the project, the website and social media of the university and through social media of an organization for gender minority individuals). Moreover, the established networks of the researchers and experts by lived experience were utilized to recruit potential participants. Recruitment took place between April and June 2023. A total of eight participants were included, originating from America (*n*=4), Europe (*n*=3) and Russia (*n*=1). Two participants were recruited via our networks and six were recruited via advertisements. Self-reported gender identities included transgender man (*n*=5), transgender woman (*n*=1), non-binary (*n*=1) and non-disclosed (*n*=1). Sexual orientation included bisexual (*n*=6) and homosexual/gay (*n*=2). All had an educational level of college/university. Time spent in Sweden ranged between two and seven years. Median age was 29 years (range: 25–49).

### Data collection

Individual semi-structured interviews were conducted by the first and last authors, both nurse-midwives and senior researchers. The interview guide included the main questions ‘How has your situation been since you came to Sweden?’, ‘What kind of support have you experienced?’, ‘How have you experienced the contact with and the treatment by health services?’ and ‘How can the support for gender minority forced migrants be further improved?’. Follow-up questions were asked as needed. The interview guide was constructed by the research team before data collection. Based on participant preferences, interviews were conducted either face-to-face (*n*=1) or digitally via a video conferencing tool (*n*=7). Median interview length was 52 min (range: 22–78 min). Before each interview, participants were provided with oral and written information about the study and asked about background characteristics. Interviews were audio recorded and transcribed verbatim in de-identified documents.

### Data analysis

The material was analysed with systematic text condensation, a descriptive and exploratory strategy for thematic cross-case qualitative analysis [17]. The iterative analytic procedure involved four steps: (i) identifying preliminary themes, (ii) identifying and sorting meaning units into groups and subgroups, (iii) producing condensates and identifying illustrative quotes and (iv) synthesizing results and generating category headings. The analysis was guided by the levels presented in the social ecological model. Two experts with lived experience of forced migration and one clinical psychologist were involved in all steps of the analysis in collaboration with the two researchers conducting the study. The researchers guided and scrutinized the analytic procedures. Daily meetings were arranged to oversee the progress, discuss the findings and jointly reach decisions. The research team consists of persons with diverse gender identities and sexual orientations.

## Results

Participants expressed resilience and hope about their future. Loneliness was a major issue contributing to health burdens and peer support was highly appreciated. Barriers hindering access to health services and judgemental behaviours among health professionals were described. While societal openness and safety for gender minority individuals was appreciated, participants faced an uncertain asylum process and unmet basic needs.

### Setting sails towards a new horizon: internal resilience and coping

Following a need to migrate from their country of origin, participants embarked on a journey involving both hope and uncertainty. They felt hopeful about their future and looked forward to being able to fully embrace Sweden as their home. They were eager to learn the language, study, explore new opportunities and secure employment necessary to gain permanent residency. Learning the language was seen as an essential component for adapting to the new society. Participants engaged in physical exercise and mindfulness to stay healthy. Mental distress such as depression and loneliness was alleviated by meditation, focusing on personal growth and wanting to leave the past behind.

I don’t want to think about what’s happened, I’m just trying to see of a way that I can just forget about everything that happened and proceed with my life. Move on with my life, start up a family and just keep living. [. . .] The way things are going right now, if the planning works I will embrace Sweden like my home. (Participant A)

### From loneliness to belonging: finding meaningful peer support

Fears of social rejection and of not being accepted in society impacted the lives of participants. Having lost their previous support system and lacking new meaningful relationships, participants felt lonely and wanted to find friends. However, challenges trusting new social contacts and fear of rejection made it difficult to connect with others. Loneliness was further exacerbated by living in the countryside, being undocumented, language barriers, financial constraints, lack of peer support activities, higher age, and cultural differences. It had been particularly challenging to find people who understood their perspectives as transgender, and some encountered transphobic behaviours in social interactions. Participants experienced neglect and segregation from non-migrant people, including within the wider community of gender and sexual minorities. While the Internet was central for finding and communicating with friends, online dating apps mostly offered surface-level connections. Newfound friendships were seen as lacking depth, with limited opportunities to discuss intimate topics.

Maybe the community doesn’t help you if you are a little older. There is discrimination between LGBTQ persons. If you are not younger. [. . .] After forty, it comes. Loneliness is a challenge for me. (Participant B)

Peer support was a main contributor to health and wellbeing. Participants highly appreciated emotional, informational and instrumental peer support. Activities arranged by non-governmental organizations prepared them for the asylum interviews and provided valuable emotional relief while they awaited a decision on their asylum claim. Community involvement with peers led to the formation of new and strong relationships, enhanced a sense of belonging and reduced loneliness. Support networks helped participants feel understood and accepted, empowering them and encouraging self-acceptance. It helped them to understand societal structures, diverse identities and different cultures. Through peer support, participants drew strength and courage from each other. Bonding with peers also enabled them to share fears and worries in an inclusive setting, reducing anxiety and stress.

It was important to meet people and participate in different activities. [. . .] Just to walk together with someone and know that you don’t have to pretend and not expect anything and not have to be careful to say anything out of line, that was important. And something new. (Participant C)

### The importance of accessible and affirming support in healthcare

Participants perceived the healthcare system as biased towards the needs of persons born in Sweden and failing to address the needs of gender minority forced migrants. Challenges in accessing health services were described, including difficulties booking appointments and needing to wait long periods for visits. When healthcare was accessed, some professionals had been experienced as insensitive, unfriendly and disinterested. Participants described feeling dismissed or discriminated against by professionals and others at the hospital, which hindered them from discussing their concerns and made them feel embarrassed. Related to this, some participants felt apprehensive about the utilization of interpreters, worrying about being judged by the interpreter and the risk of misinterpretations.

[Health professional] condemned me like for being a trans [. . .] I felt very bad, and I felt like, one thing I don’t like, it’s one talking to me in a rude manner, especially in public spaces. So, this happened and there were people there. I felt really embarrassed. Embarrassed and. . . I don’t know, but I strongly believe my gender and my race also made him react to me that way. (Participant D)

Participants also expressed gratefulness over the healthcare received in Sweden. They appreciated professionals who were interested in their health and who displayed warm, welcoming, non-judgemental and empathetic behaviours. Establishing trust, client safety, continuity of care and showing competence in diversity as well as the health of gender diverse people of colour were considered essential in clinical settings. Participants not confident conversing in Swedish appreciated when interpreter services were used, given that it involved respectful language and adhered to confidentiality. To increase safety and client comfort, participants suggested using visual cues to signal openness (e.g. PRIDE flags), inclusive language, respecting pronouns and providing continued training of health professionals.

For the healthcare providers, I’ve actually had some wonderful experience to work with them. From care to actually getting the treatment and support that I need, has been a tremendous experience so far. And I feel the healthcare system is doing a lot in educating healthcare professionals on how to handle patients irrespective of their conditions. (Participant E)

### Facing society’s cold shoulder whilst appreciating newfound safety

Participants described intersectional discrimination and exclusion within the broader society and the wider community of gender and sexual minorities. The asylum process had been a main challenge, including an emotionally draining lengthy waiting period in uncertainty. Participants feared what would happen if they were denied asylum and deported. Simultaneously, they worried about their future living conditions if they were to be granted asylum. Struggling with limited financial resources, participants had insufficient money for necessities like food and transportation. Moreover, they faced challenges in finding safe accommodation. The pressure to learn the language and establish themselves in society further contributed to mental health burdens. Several experienced challenges in securing employment to gain permanent residency and felt forced to stay in stressful work environments.

What have been the most challenging for me since I came to Sweden is that challenging aspect of getting some work done. Getting some work done, getting new work and accommodation. Accommodation has really been one of the challenging aspects. (Participant F)

Despite the societal challenges, participants nevertheless appreciated Sweden as a generally open society towards gender and sexual minorities. Some had been placed in asylum accommodations for gender and sexual minority forced migrants, which provided a sense of reassurance and safety among peers. Participants also felt a sense of safety and calm at their workplace, with less risk of threats or discrimination. Some noted a generational difference within the community, experiencing younger individuals as showing more openness and access to information.

Sweden, it is pretty safe for me. I felt, it is rather calm. I’m not in any unsafe situation here. (Participant B)

## Discussion

Loneliness is acknowledged as a major public health concern [18] and a contributor to psychological distress among refugees [19]. Our participants described significant challenges finding meaningful social contacts and loneliness had a major impact on health and wellbeing. Importantly, our participants described intersectional discrimination and social exclusion based on multiple converging identities. These findings align with the intersectional minority stress model, which proposes that health is impacted when people are marginalized based on multiple grounds [12]. Intersectionality presents a framework relevant for the health and wellbeing of gender minority forced migrants. One prior study investigating intersectional aspects of sexual minority individuals suggests higher levels of mental health burdens among those exposed to prolonged structural stigma, and, further, that stressful reactions may wane over time when exposed to supportive structures [11]. Our study adds to these findings, calling attention to the need for additional research exploring intersectional perspectives on the health and wellbeing of gender minority forced migrants.

Previous research about the wider community of sexual and gender minority forced migrants, based on cisgender gay men in non-Nordic settings, echoes our findings about the benefits of peer support [15]. Peer support, defined as a mutual supportive interaction between two persons that consider themselves as equal, has the potential to offer emotional relief in safe and allowing settings [20]. Our results suggest that this can be particularly important for gender minority forced migrants. Some studies based on refugees [21] and gender minority individuals [22] suggest that peer support interventions alleviate mental health burdens. However, few, if any, intervention studies have evaluated the effectiveness of peer support among gender minority forced migrants. More research is needed to understand how peer support can be utilized in healthcare and social care, to improve the health and wellbeing of this underserved population.

Reviews call attention to the unmet health needs of the broader population of forced migrants and the need to address barriers hindering their access to healthcare [7,23]. Our participants came across similar barriers and expressed that the system is biased towards the needs of the non-migrant population. Once healthcare was accessed, our participants faced non-affirming behaviours and discrimination. Fears and worries of encountering such behaviours resulted in hesitation to seek healthcare. Previous studies suggest that forced migrants [7,24] and transgender persons [25] experience discriminatory practices in healthcare. In line with our findings, one quantitative study found that transgender persons holding an additional marginalized identity are at increased risk of discrimination in healthcare [26]. Our participants had encountered transphobic attitudes and suggested a need for competence development for health professionals. Previous studies highlight that health professionals lack knowledge about the health of gender minorities [27] and that more content about the needs of gender minority forced migrants is necessary in education [28]. We encourage more research about discrimination in healthcare and how to promote an affirming clinical approach. Based on our findings, essential components of care could include non-judgemental, respectful and empathetic support that ensures safety and confidentiality.

There are methodological limitations related to the trustworthiness of this study. A combination of convenience, purposeful and snowball sampling was used to include eight participants. All participants were highly educated and most were transgender men. The limited sample size and lack of diversity regarding educational level may be an expression of a hesitancy to participate based on mistrust towards research or confidentiality fears [29]. The high educational level and time spent in Sweden could have influenced the data. This needs to be considered when interpreting the findings. While this study provides important findings about an underrepresented population, we acknowledge the limitations related to the sample size and transferability. On the other hand, the data was rich and the number of participants corresponds to the recommended levels for systematic text condensation [17]. Two Swedish-born researchers collected the data, and their non-migrant backgrounds may have influenced the material. An interview guide with open-ended questions was used, but it was possible that different interviewers would uncover additional information. The modality of the interview was based on participant preferences, and it is probable that participants chose to be interviewed in the way that they felt most comfortable with. Nevertheless, we acknowledge that use of digital interviews could have influenced the richness of the data. To approach the data from diverse perspectives, two researchers analysed the data in close collaboration with two experts by lived experience and one clinical psychologist. Limited research has been conducted collaboratively with gender and sexual minority forced migrants. We argue that our analytic approach enriched and nuanced the findings. Researcher bias always needs to be considered when interpreting the findings of qualitative studies. We encourage more research that will complement our findings.

## Conclusion

Gender minority forced migrants show resilience and appreciate the newfound societal safety. However, they find themselves in the margins of society and encounter various multi-layered challenges. Loneliness is a public health concern that could be addressed through peer support, which is highly desired and valued. Ensuring access to affirming health services should be a prioritized area for researchers, professionals, stakeholders and policy-makers.
